# Clinical Geriatrics as a Team Sport: Fostering Interprofessional Experiences in the Community

**DOI:** 10.1093/geroni/igab046.3005

**Published:** 2021-12-17

**Authors:** David Picella, Diana Woods

**Affiliations:** Azusa Pacific University, Azusa, California, United States

## Abstract

Team-based care is necessary to provide better healthcare outcomes for the complex needs of older adults. Shared clinical learning experiences prepare practitioners to work in collaborative partnership to achieve optimal outcomes. To promote collaborative partnership, we established interprofessional community based clinical experiences with older adults at home, in assisted living and in skilled nursing facilities. One nurse practitioner faculty member was paired with 2 students for each clinical experience day. Initially these were face-to-face encounters, however, with the onset of COVID-19, all high-risk encounters were converted to a virtual modality. The clinical encounters focused on the Age Friendly Model (4M). Post clinical discussions and recommendations focused on interprofessional treatment plans. A REDCap(TM) survey was completed by all student participants for program evaluation. Of the 14 surveys sent, 11 were completed; 10 (77%) females; 3 (23%) males; 7 (50%) family practitioner students; 7 (50%) adult-gerontology nurse practitioner students. Four had previous home health experience (14%), and 10 had none (86%). 4M Likert scale (1-5) means were “what matters” = 4.27, medications = 4.18, mentation = 4.09, and mobility = 4.09. Students found the overall experience valuable (mean = 4.27). Of 11 students, 3 (27%) were involved telehealth experiences. Students found real community based clinical experiences to be very enlightening, offering a different perspective, and altering their appreciation for the everyday life of the older adult. Future plans include adding social work and physical therapy students to these clinical experiences to enhance interprofessional education.

